# Unwarranted regional variation in vertebroplasty and kyphoplasty in Switzerland: A population-based small area variation analysis

**DOI:** 10.1371/journal.pone.0208578

**Published:** 2018-12-10

**Authors:** Claudia Scheuter, Maria M. Wertli, Alan G. Haynes, Radoslaw Panczak, Arnaud Chiolero, Arnaud Perrier, Nicolas Rodondi, Drahomir Aujesky

**Affiliations:** 1 Division of General Internal Medicine, Bern University Hospital, Inselspital, University of Bern, Bern, Switzerland; 2 Clinical Trials Unit, University of Bern, Bern, Switzerland; 3 Institute of Social and Preventive Medicine, University of Bern, Bern, Switzerland; 4 Institute of Primary Health Care (BIHAM), University of Bern, Bern, Switzerland; 5 Department of Epidemiology, Biostatistics, and Occupational Health, McGill University, Montreal, Canada; 6 Division of Internal Medicine, Rehabilitation and Geriatrics, Geneva University Hospitals, Geneva, Switzerland; National Yang-Ming University, TAIWAN

## Abstract

**Background:**

Percutaneous vertebroplasty (VP) and balloon kyphoplasty (BKP) for treating painful osteoporotic vertebral fractures are controversial.

**Objective:**

We assessed the regional variation in the use of VP/BKP in Switzerland.

**Methods:**

We conducted a population-based small area variation analysis using patient discharge data for VP/BKP from all Swiss hospitals and Swiss census data for calendar years 2012/13. We derived hospital service areas (HSAs) by analyzing patient flows, assigning regions from which most residents were discharged to the same VP/BKP specific HSA. We calculated age-/sex-standardized mean VP/BKP-rates and measures of regional variation (extremal quotient [EQ], systematic component of variation [SCV]). We estimated the reduction in variation of VP/BKP rates using negative binomial regression, with adjustment for patient demographic and regional socioeconomic factors (socioeconomic status, urbanization, and language region). We considered the residual, unexplained variation most likely to be unwarranted.

**Results:**

Overall, 4955 VP/BKPs were performed in Switzerland in 2012/13. The age-/sex-standardized mean VP/BKP rate was 4.6/10,000 persons and ranged from 1.0 to 10.1 across 26 HSAs. The EQ was 10.2 and the SCV 57.6, indicating a large variation across VP/BKP specific HSAs. After adjustment for demographic and socioeconomic factors, the total reduction in variance was 32.2% only, with the larger part of the variation remaining unexplained.

**Conclusions:**

We found a 10-fold variation in VP/BKP rates across Swiss VP/BKP specific HSAs. As only one third of the variation was explained by differences in patient demographics and regional socioeconomic factors, VP/BKP in the highest-use areas may, at least partially, represent overtreatment.

## Introduction

Symptomatic vertebral fractures are common in the elderly and potentially result in pain, deformity, disability, and substantial costs [1, 2]. Treatment options include conservative management with analgesia, bed rest, and physical therapy, and invasive vertebral augmentation procedures, such as percutaneous vertebroplasty (VP) and balloon kyphoplasty (BKP) [3]. In VP, bone cement is injected into a collapsed vertebral body, whereas BKP uses an inflatable balloon to create vertebral body expansion before the injection of cement [4]. Following evidence from open-label randomized and uncontrolled studies that VP/BKP rapidly reduce pain in osteoporotic vertebral fractures and rarely lead to severe complications (<2%) [5–8], VP/BKP were quickly adopted into clinical practice, with a 741% increase in VP/BKP rates in the U.S. from 2001 to 2008 and annual costs of $672 million [9, 10].

In 2009, two placebo-controlled randomized trials demonstrated that VP was not superior in alleviating pain than a sham procedure in up to 12 months old painful osteoporotic fractures [11, 12]. As a consequence, the American Academy of Orthopedic Surgeons [13] recommended against the use of VP for osteoporotic vertebral fractures, and considered BKP as an option with the caveat of limited evidence. On a policy level, the Australian government revoked public funding for VP/BKP in 2011 [14]. Others, such as the National Institute for Health and Care Excellence, recommended restricted use in patients with ongoing pain despite optimal management [15]. Presumably as a result of these trials, the VP rates decreased by 63% and the BKP rates by 10% from 2004 to 2014 in the U.S [16]. However, to which patient VP or BKP should be recommended remained highly controversial. This controversy was fueled by the open label VERTOS II trial [6] and the sham procedure controlled VAPOUR trial [17] that showed a decreased pain level in favor of VP in patients with acute fractures with a pain duration of <6 weeks, but with controversial opinions on the sham procedure that differed from previous trials. In contrast to these positive results, the sham procedure controlled VERTOS IV trial showed no benefit of VP compared to a sham procedure in painful osteoporotic vertebral fractures of <9 weeks’ duration [18].

Although the Swiss Medical Board recommended to use VP only in patients with ongoing pain and after interdisciplinary consensus in 2011 [19], Swiss health insurers continued to cover the costs of all vertebral augmentation procedures, and current VP/BKPs rates across Swiss regions are unknown. As geographical variations in controversial preference-sensitive procedures, such as VP/BKP, may represent rather differing physician opinions than differences in medical need or patient preferences,[20–24] we examined regional variations in the use of VP/BKP in Switzerland during 2012/2013. In disorders for which surgery is almost universally recommended, such as hip fracture or appendicitis, low variation in procedure rates across geographical areas has been observed [20]. A more than three-fold variation in procedure rates has been suggested to be mainly explained by differences in practice style or medical discretion [24]. Assuming a similar geographic distribution of osteoporotic vertebral fractures and patient preferences, regional variations not explained by differences in population characteristics would most likely represent unwarranted variation.

## Materials and methods

### Data sources

We conducted a population-based small area variation analysis based on routinely collected patient discharge data from all Swiss acute care hospitals and Swiss census data for calendar years 2012 and 2013. With the introduction of a diagnosis related group reimbursement system (SwissDRG) in 2012, hospital payments in Swiss acute care hospitals are linked to the provision of accurate diagnoses/interventions, leading to an unprecedented level of data quality. Swiss hospitals are legally obligated to provide the Swiss Federal Statistical Office (SFSO) an anonymized, standardized data set for each hospital discharge. These data are centrally stored in the Swiss Hospital Discharge Masterfile hosted at the SFSO. Recorded variables include patient age, sex, nationality, insurance type, the principal and up to 49 secondary diagnostic codes based on the International Classification of Diseases, 10^th^ revision, German Modification (ICD-10-GM), up to 100 procedure codes based on the Swiss Classification of Operations (CHOP, an adaptation of the U.S. ICD-9-CM volume 3 procedure classification), and the admission type. The area of patient residence and hospital location within one of 705 Swiss MedStat regions are recorded. MedStat regions represent aggregated ZIP-codes regions, with a population size of approximately 10,000 persons per region [25]. The SFSO reviews data integrity and completeness by means of a specifically designed software. Overall, the Swiss Hospital Discharge Masterfile covered 100% of discharges during 2012/2013 [26, 27].

Using registry-based 2012 census data from the SFSO, we determined the number of inhabitants, age- and sex distribution, main language (Swiss German, French, or Italian), socioeconomic status (combining neighborhood information on rent, education, occupation, and crowding), and the level of urbanization (urban, peri-urban, mixed, rural) for each MedStat region. The SFSO defines the level of urbanization using a multistep approach based on spatial (density of resident and working population, overnight stays in hotels) and functional criteria (commuter travel patterns) [28]. Because our study was based on anonymized administrative data, it was exempted from ethics committee approval according to the Swiss Human Research Act.

### Derivation of Swiss Hospital Service Areas

Switzerland has compulsory basic health insurance coverage, with voluntary semiprivate and private insurance plans covering additional services. Although Swiss hospital care is primarily organized based on 26 geographic regions, the cantons, patients may utilize hospital services outside their canton of residence and the use of cantons as a unit of observation may skew procedure rates. We therefore used Wennberg and Gittelsohn’s approach to derive Swiss Hospital Service Areas (HSAs) [29], which takes into account patient flow patterns. Because the procedure counts for VK/BKP alone were too low, we used the data from patients discharged for one of eight predefined preference-sensitive procedures during 2012/2013 (VP/BKP, hip/knee replacement, cholecystectomy, hysterectomy, prostate surgery, coronary artery bypass grafting, and percutaneous closure of a patent foramen ovale) to derive Swiss HSAs.

We initially identified 2,318,595 patient discharges from 155 Swiss acute care hospitals during calendar years 2012 and 2013. Of these, 153,678 patients (6.6%) were discharged for one of the eight predefined preference-sensitive procedures. After the exclusion of 3117 discharges with residence outside Switzerland or missing/invalid MedStat region and 159 discharges aged <18 years, we analyzed the flows of 150,402 discharges (98%). We assigned MedStat regions from which the highest proportion of residents was discharged to the same HSA (plurality rule) [30], which yielded 74 potential HSAs. As VK/BKP are specialized procedures that are not available in every hospital, we repeated the same process and aggregated the 74 initial HSAs into 26 larger VP/BKP specific HSAs. We then drew visual maps of the 26 final VP/BKP specific HSAs using Geographical Information System (GIS)-compatible vector files for MedStat regions. Finally, we visually examined the borders of each HSA to ensure map readability and reassigned MedStat regions to another HSA in two cases.

### Study population

We used the Swiss Hospital Discharge Masterfile to identify patient discharges with a specific Swiss Classification of Operations procedure code for VP (81.65.00, 81.65.10–13, 81.65.99) or BKP (81.66.00, 81.66.10–13, 81.66.99) from all Swiss acute care hospitals during 2012/2013. Overall, we identified 4946 adult discharges with specific codes for VP/BKP and complete MedStat region information. Because pathologic fractures due to vertebral metastases or multiple myeloma may be appropriate indications for VP/BKP,[31, 32] we excluded 387 (9%) discharges with ICD-10-GM codes for metastatic cancer or multiple myeloma (ICD-10 codes C77.x–C80.x and M82.0), leaving a final study population of 4559 patient discharges. We described the comorbid burden of the final study population using a modified Elixhauser Comorbidity Index that can be condensed into a single numeric score summarizing the overall disease burden (range: −19 to +89 points) [33].

### Measures and determinants of variation

We calculated unadjusted and age-/sex-standardized VP/BKP procedure rates per 10,000 persons for each VP/BKP-specific HSA using procedure counts and 2012 census data for the adult Swiss population [34]. We used direct standardization with 5-year age bands from 15 to ≥95 years. As the prevalence of osteoporosis is highest in the elderly population [1], we also determined unadjusted and age-/sex-standardized rates of VP/BKP in persons aged ≥65 years. To examine the variation in procedure rates across VP/BKP specific HSAs, we determined the extremal quotient (EQ, defined as the highest divided by the lowest procedure rate). While the EQ is an intuitive measure of variation, it is prone to distortion by extreme values [24]. We also calculated the coefficient of variation (CV), defined as the ratio of the standard deviation of the procedures rates to the mean rate, and the systematic component of variation (SCV) [24, 35]. Although less intuitive, the SCV represents the non-random part of the variation in procedure rates while reducing the effect of extreme values [24, 35, 36]. The SCV is derived from a model that recognizes the differences in rates across areas and the random (by chance) variation within each area’s true rate. In clinical disorders for which surgery is almost universally recommended, such as hip fracture or appendicitis, a low variation across HSAs with an SCV of less than 3.0 was found [20]. For example, the SCV for hip fracture was 1.8 in Switzerland indicating a very low variation [37]. In diseases where various treatment options are available, wider variation in procedure rates have been observed [20]. Individual physicians’ preferences and beliefs are the most important reasons for the regional variation and may at least partially indicate unwarranted variation. A SCV >10 is considered indicative of a very high variation [24, 36]. It has been suggested that SCVs >5 are largely due to differences in practices styles or medical discretion [24, 36].

Because differences in illness incidences and socioeconomic factors are possible causes of variation [24], we explored potential determinants of warranted (need-based) variation at the HSA-level: age, sex, socioeconomic status, level of urbanization, and language region (a proxy for cultural differences). The socioeconomic status of each HSA was calculated using the median value of the Swiss Neighborhood Index of socioeconomic position (SSEP) of all areas within the HSA [38]. The SSEP consists of four domains (median rent per m^2^, proportion of households led by a person with no/low education, proportion headed by a person in manual/unskilled occupation, and mean crowding, all on the neighborhood level). The SSEP varies between 0 (worst) and 100 (best) and correlates well with mortality [39].

We used progressively adjusted multilevel negative binomial regression to model the procedure rates in each HSA using 5-year age- and sex-strata. Model 1 was an intercept-only model. Model 2 was adjusted for age and sex. Model 3 was additionally adjusted for SSEP, level of urbanization, and language region. We depicted the variation in HSA rates as average predicted VP/BKP rates per 10,000 per HSA derived from the multilevel regression models. To assess the impact of patient demographics and socioeconomic factors on the VP/BKP rates, we used the same progressively adjusted models. We expressed the impact of patient demographics and socioeconomic factors on VP/BKP rates as incidence rate ratios (IRRs), defined as the VP/BKP rate in the defined category (e.g., women) relative to the estimated VP/BKP rate in the reference category (e.g., men). We also determined the percentage reduction in procedure variation across the 26 VP/BKP specific HSAs by examining the variance of the random intercept. We considered the residual, unexplained variance of the fully adjusted model a proxy for unwarranted variation. Statistical analyses were performed using Stata version 14.2 (StataCorp, College Station, TX, USA) and R statistical software version 3.2.3 [40].

## Results

### Characteristics of Swiss Hospital Service Areas and the study population

**Fig 1:**
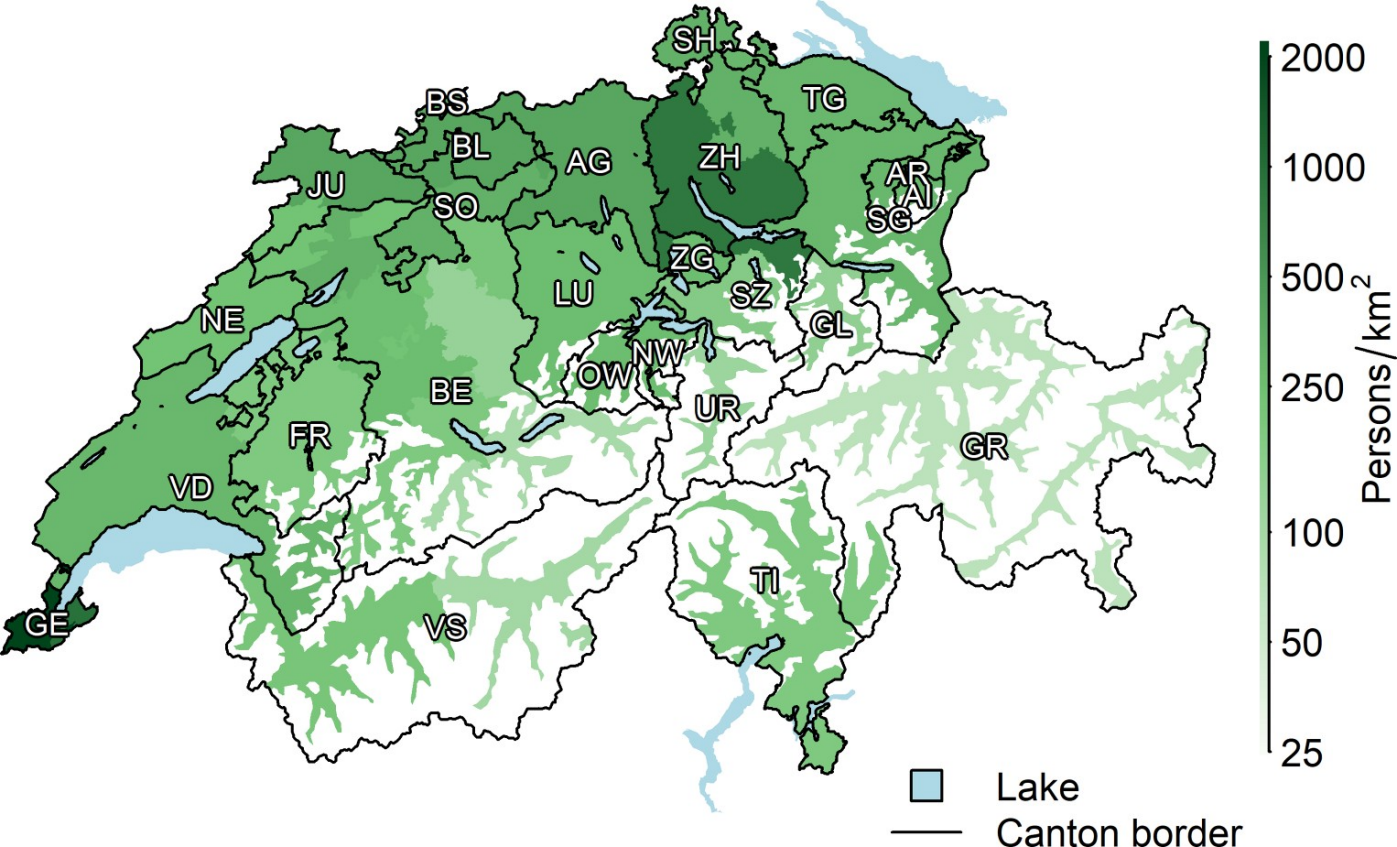
Map of the 26 vertebroplasty/kyphoplasty specific HSAs with cantonal borders and population density. Abbreviations: AG = Aargau; AI, Appenzell-Innerrhoden; AR = Appenzell-Ausserrhoden; BE = Bern; BL = Basel-Land; BS = Basel-Stadt; FR = Fribourg; GE = Geneva; GL = Glarus; GR = Grison; JU = Jura; LU = Lucerne; NE = Neuchâtel; NW = Nidwalden; OW = Obwalden; SG = St. Gallen; SH = Schaffhausen; SO = Solothurn; SZ = Schwyz; TG = Thurgau; TI = Ticino; UR, Uri = VD = Vaud; VS = Valais; ZG = Zug; ZH = Zurich. HSAs are represented by different colors.

**Fig 1** shows the geographic boundaries of the 26 VP/BKP specific HSAs and the population density. The mean number of acute care hospitals per HSA was 12 (range 2–54). The mean population size per HSA was 307,211 persons (range 35,466–1,300,784), with a mean population density of 335 persons per km^2^ (range 28–2221), and a median SSEP of 62 points (range 51–78). Overall, 19 HSAs were located in the Swiss German-speaking, 6 in the French-speaking, and 1 in the Italian-speaking part of Switzerland.

Of the 4559 adult patients discharged with VP/BKP, 2894 (63.5%) underwent VP, 1278 (28.0%) BKP, and 387 (8.5%) both procedures. The majority of patients were older (mean age 73 years), women (71%), and Swiss nationals (93%) (**Table 1**). Overall, 80% of patient discharges had one or two vertebral bodies treated. Thirty-four patients (1%) died during hospitalization.

**Table 1:** Characteristics of the study population (N = 4559) undergoing vertebroplasty or kyphoplasty during calendar years 2012 and 2013.

Characteristics	n (%) or mean±SD
Demographics	
Age, years*	73±12
Female sex	3233 (71)
Swiss nationality	4259 (93)
Insurance status	
Basic	2795 (61)
Semi-private	1138 (25)
Private	626 (14)
Reason for admission	
Emergency	1733 (38)
Elective	2706 (59)
Other/unknown	120 (3)
Elixhauser Score, points	3±5
Vertebral bodies treated, no.	
1	2592 (57)
2	1039 (23)
3	639 (14)
4+	551 (12)
Not specified	33 (1)

Abbreviations: SD = standard deviation.

*The mean age was calculated using the midpoint of 5-year age intervals.

### Variation in procedure rates across health service areas

**Fig 2:**
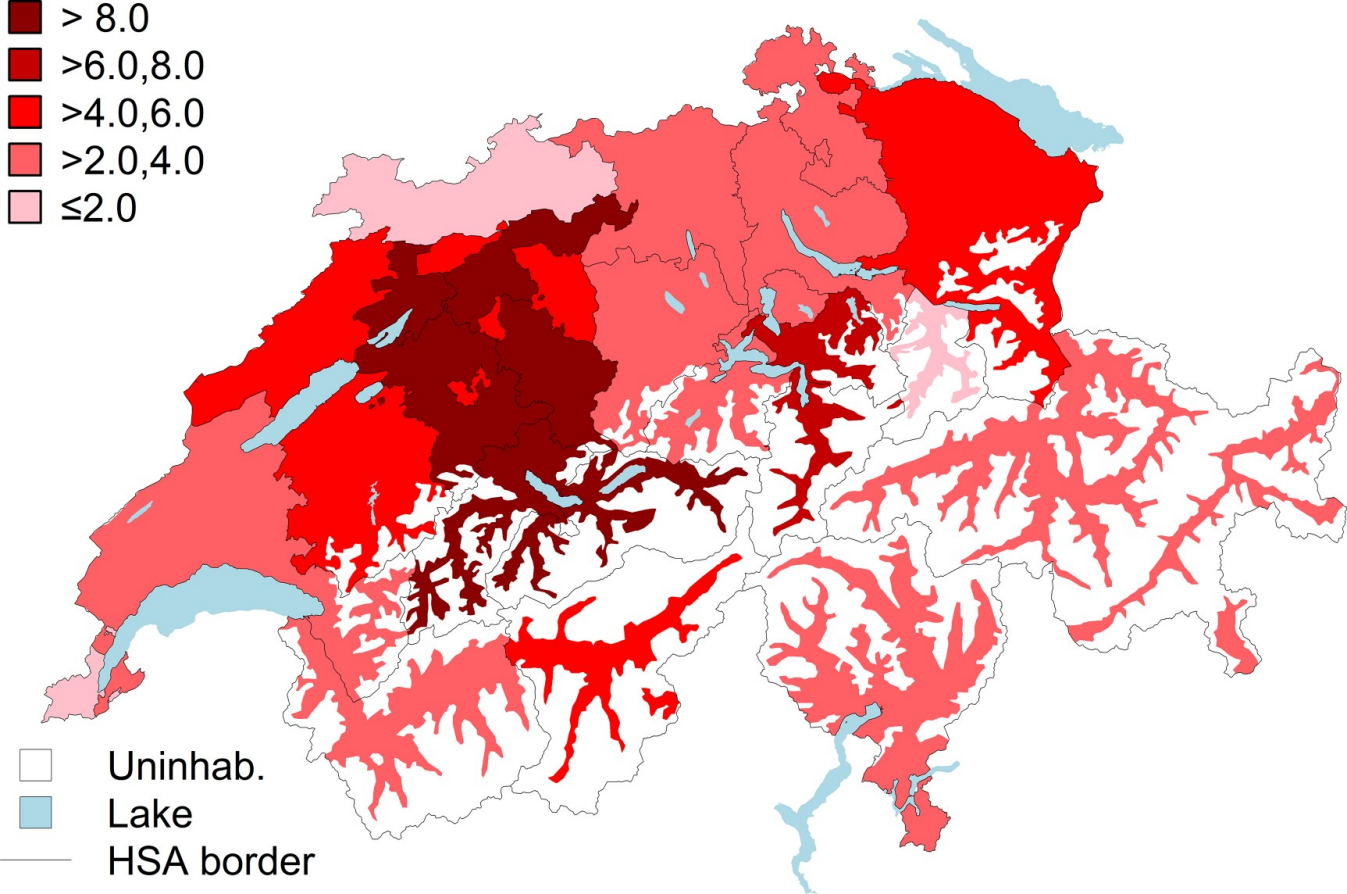
Age- and sex-standardized vertebroplasty/kyphoplasty procedure rates per 10,000 persons across procedure specific hospital service areas. Abbreviations: uninhab. = uninhabited area; HSA = hospital service area.

The mean unadjusted VP/BKP rate across VP/BKP specific HSAs was 5.2 procedures (range 1.2–11.9) per 10,000 persons. The mean age-/sex-standardized procedure rate was 4.6 (1.0–10.1) per 10,000 persons. In patients aged ≥65 years, the mean unadjusted and age-/sex-standardized VP/BKP rates across HSAs were 17.9 procedures (range 3.7–37.5) and 17.4 (range 3.6–37.4) per 10,000 persons, respectively. Crude and age-/sex-standardized procedure rates for each HSA are shown in the Supplement (**S1 Table)**. Ranges of age-/sex-standardized procedure rates across HSAs are shown in **Fig 2**. We found the highest procedure rates (>6.0 per 10,000 persons) in HSAs located in the greater Bern area, Uri, and Schwyz, and the lowest rates (0–2.0 per 10,000 persons) in Zurich, Jura, Basel, Glarus, Geneva, and the western Valais. The EQ was 10.2, the CV 0.59, and the SCV 57.6, indicating a very high variation across HSAs.

### Determinants of variation in procedure rates

**Fig 3:**
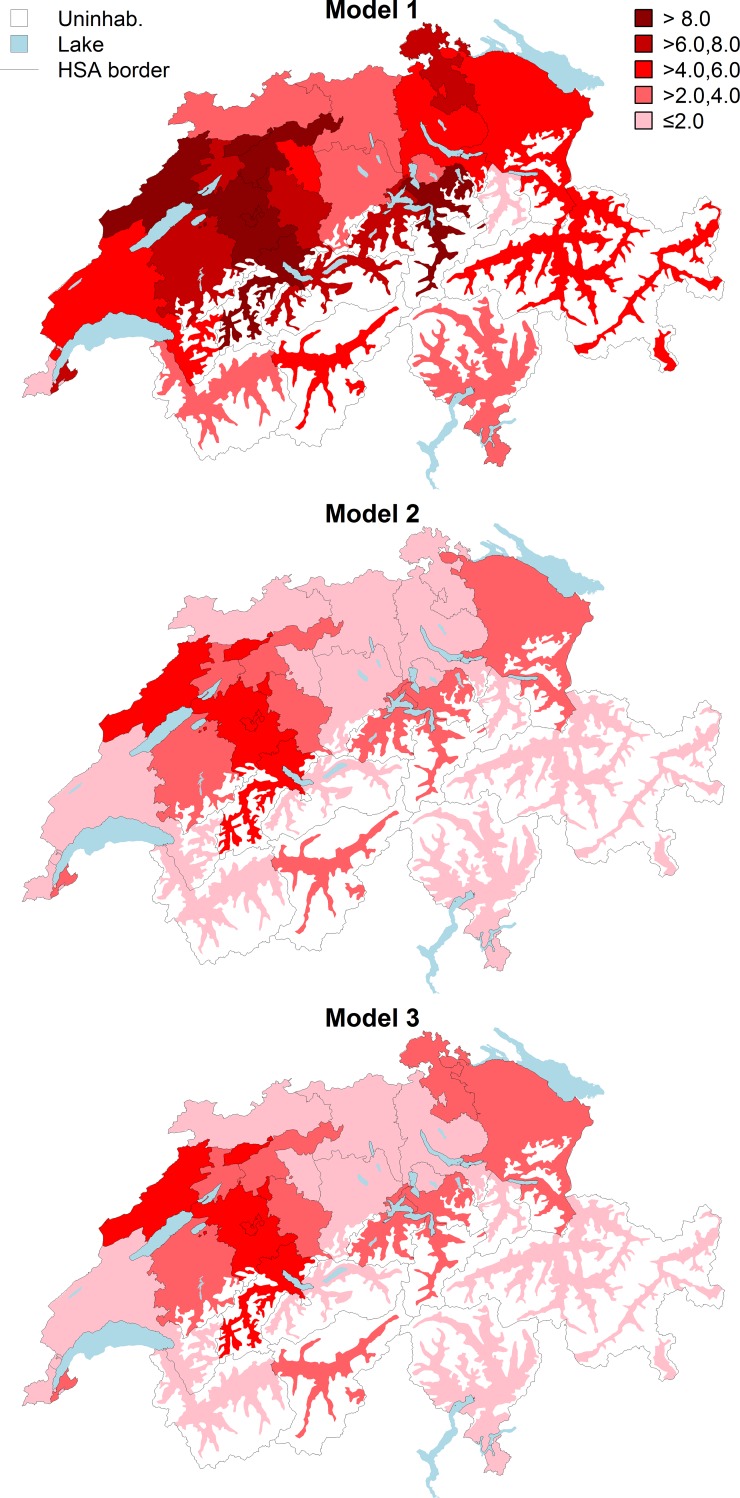
Map of the average predicted vertebroplasty/kyphoplasty rates per hospital service area derived from models with progressive adjustment. Abbreviations: uninhab. = uninhabited area; HSA = hospital service area. Average predicted VP/BKP rates for each HSA are shown as red-scale categories varying from 0–2 to >8 procedures per 10,000 persons. Model 1: Unadjusted. Model 2: Age- and sex-adjusted. Model 3: Additional adjustment for the median Swiss Neighborhood Index of Socioeconomic Position, level of urbanization, and language region.

**Fig 3** shows the map of the average predicted VP/BKP rates in the progressively adjusted models for each VP/BKP specific HSA. In general, adjustment for age and sex resulted in a reduction of the predicted average VP/BKP rates in all HSAs, albeit to a varying extent. The predicted average VP/BKP rates decreased from >8 to >2–4 procedures per 10,000 persons in several HSAs with the highest unadjusted rates (Uri and Schwyz) or from >6–8 to 0–2 procedures per 10,000 persons (Schaffhausen and the northern part of Zurich). The decrease was less impressive in other high-volume areas (Bern and Neuchâtel), where predicted average VP/BKP rates decreased from >8 to >4–6 per 10,000 persons. Additional adjustment for socioeconomic/cultural factors did not change the average predicted VP/BKP rates in most regions, except for Schaffhausen and the northern part of Zurich, where adjustment led to increased predicted average rates (from 0–2 to >2–4 per 10,000 persons).

Age and sex were the main determinants of procedure variation across VP/BKP specific HSAs (**Table 2**). For each 5-year increase in age, the procedure rate increased by 8% (IRR 1.08, 95% CI 1.08–1.09). Women had a 59% higher procedure rate than men (IRR 1.59; 95% CI, 1.40–1.80). Despite resulting in a reduction in variance amongst HSAs, SSEP, urbanization, and language region were not statistically significantly associated with procedure rates.

**Table 2:** Determinants of variance in the incidence rates of vertebroplasty/kyphoplasty across procedure specific hospital service areas.

	Model 1*	Model 2†	Model 3‡
	Incidence rate ratio (95% CI)§		
Age, per 5 years	-	1.08 (1.08–1.09)	1.08 (1.08–1.09)
Sex	-		
Male		1 (reference)	1 (reference)
Female		1.59 (1.40–1.80)	1.59 (1.40–1.80)
Language region	-	-	
German			1 (reference)
French			0.88 (0.51–1.51)
Italian			0.66 (0.19–2.33)
Urbanization	-	-	
Urban			1 (reference)
Periurban			0.69 (0.32–1.47)
Mixed			0.79 (0.32–1.97)
Rural			0.58 (0.26–1.30)
Median SSEP, per point	-	-	0.98 (0.92–1.03)
Intercept varianceII	0.43 (0.21–0.88)	0.33 (0.18–0.61)	0.29 (0.16–0.54)
Reduction in variance		22.8%	32.2%

Abbreviations: CI = confidence interval; SSEP = Swiss Neighborhood Index of socioeconomic position.

*Model 1: unadjusted.

†Model 2: age- and sex-adjusted.

‡Model 3: additional adjustment for socioeconomic factors (SSEP, level of urbanization, and language region).

§VP/BKP rate in the defined category relative to the VP/BKP rate in the reference category. For instance, an incidence rate ratio of 1.59 indicates a 59% higher VP/BKP rate in women than in men.

IIExpresses the variance in VP/BKP rates from the national mean.

Compared to the unadjusted model, adjustment for age/sex resulted in a 22.8% reduction in the variance of VP/BKP rates across the 26 HSAs relative to the national mean. With further adjustment for socioeconomic/cultural factors the total reduction in variance increased to 32.2%, with the larger part of the variation remaining unexplained.

## Discussion

Our analysis demonstrates a 10-fold variation of VP/BKP across 26 VP/BKP specific HSAs. Only about one third of the variation in procedure rates can be explained by differences in age, sex, socioeconomic status, level of urbanization, and language region. As procedure variation is unlikely to be due to regional differences in clinical need or patient preferences [24], the larger portion of the observed variation is most likely unwarranted.

Our results demonstrate that clinical use of VP and BKP was very heterogeneous across Switzerland. Because patient needs and preferences are unlikely to differ across HSAs, the variation in VP/BKP across VP/BKP specific HSAs is mostly likely to be determined by differing practices of relatively small groups of local physicians. Practice variation is related to the degree of professional uncertainty and consensus concerning the best treatment [21]. Differences among physicians in their belief in the efficacy of a specific treatment contributed substantially to the observed variation in rates of use [20, 21, 35]. Although higher quality evidence from two trials demonstrated no benefit from VP compared to sham procedures, the majority of subsequent open-label trials showed a superiority of VP in reducing pain compared to conservative treatment [6, 7, 41–43]. Thus, the variation in VP/BKP rates in Switzerland during 2012/13 may primarily reflect the professional controversy about the benefits of VP/BKP. Since 2014, the controversy was further fueled by recommendations of the American Society of Interventional Radiology and others to perform early VP in patients with immobilizing or intolerable pain persisting >24 hours and the sham procedure-controlled VAPOUR trial demonstrating a decreased pain level in favor of VP in patients with acute fractures with a pain duration of <6 weeks [8, 17]. In contrast to these findings the sham procedure-controlled trial, the VERTOS IV trial published in 2018, found VP no more effective than a sham procedure in patients with acute fracture with a pain duration of <9 weeks [18]. A Cochrane review concluded, based on five high quality trials, that VP provided no clinically important benefits compared to sham procedures and that there was no evidence that BKP was superior to VP [44]. However, to date no trial is available that compared BKP to a sham procedure. Although mandatory reimbursement could drive the overall use of VP/BKP, reimbursement rates are similar across Swiss cantons and financial incentives cannot plausibly explain the large variation across Swiss regions. Given that the Swiss population enjoys universal health care coverage, each VP/BKP specific HSA included at least one center performing the procedure, and access to care is excellent [45], regional variations are unlikely to be explained by care access disparities.

Differences in population age and sex explained 28.3% of the variation across HSAs, with women—known to have a relatively high risk of osteoporotic vertebral fractures [1]—having a 59% higher rate of VP/BKP than men. Although poorer areas suffer higher illness rates than more affluent areas [24], rural areas have more limited access to centers offering specialized procedures [24], and more specifically for Switzerland, French/Italian speaking regions consume more health care than Swiss German speaking areas [46], additional adjustment for regional socioeconomic position, level of urbanization, and language resulted in a further variation reduction of <10% only. Thus, the socioeconomic factors considered in our study do not appear to be major influencing factors of VP/BKP rates in Switzerland.

It is impossible to determine the “appropriate” rate of a controversial procedure, such as VP/BKP. However, in light of guideline recommendations in the period between 2010 and 2013 that VP/BKP should be avoided in most patients with painful osteoporotic vertebral fractures, VP/BKP rates in the highest-use areas HSAs are likely to represent overtreatment.

The great variation in VP/BKP rates is not a unique Swiss phenomenon. The proportion of Medicare beneficiaries with spine fractures who received VP varied from 0% to >20% across counties in Pennsylvania from 2001 to 2004 [47]. Medicare data also showed a substantial regional variation, with VP/ BKP rates ranging from 4.3 in Alaska to 55 per 10,000 persons in North Dakota [48]. Our results indicate that a specific pattern of procedure variation exists across international boundaries that is independent of the national method of organizing medical care [35]. The 2012/13 VP/BKP rate in patients aged ≥65 years was similar in Switzerland and in the U.S. (18 vs. 16 per 10,000 persons) [16].

While the use of population-based data of high quality and completeness is a distinctive strength of our study, our work has also potential limitations. First, we could not explore other factors potentially responsible for practice variation, such as differences in patient preferences, the population disease burden, and the prevalence of osteoporosis. However, we have no plausible explanation why patient preferences with respect to VP, the epidemiology of osteoporosis, or other unmeasured confounders would vary across geographically close VP/BKP specific HSAs. Second, we could not examine whether regional differences in supply-side factors, such as in the density of specialists performing VP/BKP (orthopedic surgeons, radiologists, and neurosurgeons), are associated with the rate of VP/BKP. However, the large differences in VP/BKP across metropolitan areas (e.g., Bern vs. Zurich), where such specialists tend to concentrate, and the high rates in some non-metropolitan areas (e.g., Neuchâtel) indicate that the regional density of specialists performing VP/BKP might not be the only explanation for variation. Finally, we included inpatient VP/BKP only and could not examine outpatient VP/BKP because of lack of national data. However, according to a major Swiss health insurer, the vast majority of BKP was performed in hospitalized patients and the proportion of outpatient VP procedures was small (<10%).

To reduce practice variation, further high-quality evidence from placebo-controlled randomized trials is needed to better define which patients benefit most from vertebral augmentation procedures (e.g., those with acute fractures) [49, 50]. This evidence should be the basis of future guideline recommendations regarding the indication for VP/BKP. Such guidelines, if properly implemented, could help improve indication quality and guide future reimbursement decisions. Evidence suggests that financial and regulatory interventions may result in lower surgical rates [51]. While awaiting new trial evidence, patient and physician education measures may be necessary to improve informed decision making regarding the potential benefits and harms of vertebral augmentation procedures vs. conservative therapy. Physician and patient education initiatives were shown to increase knowledge and reduce surgery rates in patients with low back pain [52, 53].

## Conclusion

In conclusion, we found a 10-fold variation in VP/BKP procedure rates across Swiss regions. As the larger portion of the variation remains unexplained by demographic and socioeconomic factors and is unlikely to be explained by differences in patient preferences, regional procedure rates are mostly likely driven by differing practices of local physicians, in line with the professional controversy regarding the benefits of VP/BKP. As VP/BKP is currently not recommended in most instances of osteoporotic vertebral fracture, VP/BKP in the highest-use areas may, at least partially, represent overtreatment.

## Supporting information

S1 Table Vertebroplasty/kyphoplasty rates in hospital service areas. §academic university hospital located in the HSA.
(DOCX)
