# Supplementary material for: Unwarranted regional variation in vertebroplasty and kyphoplasty in Switzerland: A population-based small area variation analysis
Source: PLoS One. 2018 Dec 10;13(12):e0208578. doi: 10.1371/journal.pone.0208578 (PMC6287855; doi:10.1371/journal.pone.0208578)
Supplement: S1 Table — §academic university hospital located in the HSA. (DOCX) [file pone.0208578.s001.docx]

**S 1 Table: Vertebroplasty/kyphoplasty rates in hospital service areas**

| **HSA** | **Interventions** | **Crude rate** | **Standardized rate** |
| --- | --- | --- | --- |
| GE05§ | 63 | 1.18 | 0.99 |
| BS06§ | 125 | 1.57 | 1.35 |
| GL01 | 10 | 1.78 | 1.55 |
| VS17 | 64 | 1.98 | 1.65 |
| ZH06§ | 420 | 1.98 | 1.96 |
| AG07 | 256 | 2.63 | 2.48 |
| VD03§ | 280 | 2.66 | 2.50 |
| LU02 | 176 | 3.26 | 2.68 |
| GR01 | 90 | 3.37 | 2.92 |
| TI13 | 173 | 3.43 | 2.98 |
| GE06 | 34 | 3.35 | 3.04 |
| ZH72 | 143 | 3.67 | 3.14 |
| LU04 | 74 | 3.92 | 3.25 |
| SG02 | 502 | 4.01 | 3.83 |
| BE06 | 278 | 5.32 | 4.82 |
| FR02 | 235 | 5.55 | 4.96 |
| BE50 | 68 | 6.06 | 5.29 |
| VS09 | 77 | 6.48 | 5.69 |
| SZ04 | 76 | 6.44 | 5.79 |
| BE31 | 142 | 7.89 | 6.93 |
| UR02 | 41 | 8.96 | 7.09 |
| SO19 | 282 | 8.00 | 7.63 |
| BE89 | 85 | 10.34 | 8.28 |
| BE01§ | 458 | 10.49 | 8.80 |
| BE55 | 155 | 9.97 | 9.57 |
| BE75 | 252 | 11.88 | 10.12 |

§academic university hospital located in the HSA
